# Serum Neurofilament Light Chain Measurement in MS: Hurdles to Clinical Translation

**DOI:** 10.3389/fnins.2021.654942

**Published:** 2021-03-25

**Authors:** Simon Thebault, Ronald A. Booth, Carolina A. Rush, Heather MacLean, Mark S. Freedman

**Affiliations:** ^1^Department of Medicine, The Ottawa Hospital Research Institute, The University of Ottawa, Ottawa, ON, Canada; ^2^Department of Pathology and Laboratory Medicine, The Eastern Ontario Regional Laboratory Association, The Ottawa Hospital, Ottawa Hospital Research Institute, The University of Ottawa, Ottawa, ON, Canada

**Keywords:** multiple sclerosis, translation, neurofilament light, blood, biomarker

## Abstract

Measurement of serum neurofilament light chain concentration (sNfL) promises to become a convenient, cost effective and meaningful adjunct for multiple sclerosis (MS) prognostication as well as monitoring disease activity in response to treatment. Despite the remarkable progress and an ever-increasing literature supporting the potential role of sNfL in MS over the last 5 years, a number of hurdles remain before this test can be integrated into routine clinical practice. In this review we highlight these hurdles, broadly classified by concerns relating to clinical validity and analytical validity. After setting out an aspirational roadmap as to how many of these issues can be overcome, we conclude by sharing our vision of the current and future role of sNfL assays in MS clinical practice.

## Introduction

The spectrum of multiple sclerosis (MS) disease severity is broad, encompassing mild or even benign forms of the disease that may not require treatment at all (Sartori et al., 2017) to rapid progressors who accumulate irreversible worsening early-on unless drastic interventions are made (Atkins et al., 2016). Due to advancements in disease modifying therapies (DMT) for MS over the past two decades, a range of treatment options are now available, and “no evidence of disease activity” on clinical and MRI measures is a realistic treatment goal for many patients (Lublin, 2012). Following assessment of demographic, clinical and MRI features, patients identified as having more severe disease are increasingly treated with higher efficacy therapies up-front, with a lower threshold for treatment escalation upon disease breakthrough on less effective treatments (Giovannoni, 2018). However, a double-edged sword, the expanding range of higher efficacy immunosuppressive therapies is often accompanied by toxicity and iatrogenic morbidity. As such, neurologists aspire to closely titrate the minimal treatment intensity required to achieve disease control, and quickly react to breakthrough activity thereafter (Freedman et al., 2020). However, the current *status quo* of disease prognostication and monitoring in MS in routine clinical practice leaves much to be desired, lagging behind the therapeutic advancements. Clinical decision-making is still dependent on a synthesis of incomplete clinical and MRI information. Initial treatment selection remains a vaguely informed decision, based on our best assessment and patient preferences, but conflated by financial/insurance considerations and clinician preference. Once on a given therapy, subsequent escalation of therapy often lags behind the damaging disease activity that neurologists and their patients seek to prevent. Fluid biomarkers that conveniently and accurately measure and track subclinical disease activity have been long sought to fill this knowledge and practice gap (Comabella and Montalban, 2014). After decades of searching, serum concentrations of neurofilament light chain (sNfL) have emerged over the past few years as a promising candidate.

Neurofilament light chain (NfL) is the most abundant of a family of highly conserved neuron-specific structural neurofilament proteins (Fuchs and Cleveland, 1998). Although our understanding of the physiology, pathophysiology and kinetics remains incomplete, it has been known for some time using conventional assays that CSF NfL is elevated in neurological conditions that cause neuroaxonal damage, such as MS (Eikelenboom et al., 2003). In the last 5 years, with the advent of ultrasensitive single molecule detection technologies, reliable blood measurements which correlate with CSF concentrations has become possible (Kuhle et al., 2016a; Wilson et al., 2016). Although concentrations in serum and plasma are closely related, they are not interchangeable; as the majority of current evidence relates to NfL concentrations in serum (sNfL), this is the focus of this review. Analogous to the cardiologist’s troponin, the promise of a serum test of neuroaxonal damage has driven remarkable interest (Thebault et al., 2020b). Although sNfL has broader applicability, MS has become the test case for sNfL clinical utility due the unmet need for biomarkers and facilitated by extensive availability of specimens from retrospective cohorts (Thebault et al., 2021). In MS, sNfL is primarily a surrogates of inflammatory disease activity, the highest concentrations seen around the time of relapses and new MRI lesions, where sNfL trends up and then down for several months before and after (Kuhle et al., 2016b; Akgün et al., 2019). In pragmatic clinical settings outside of clinical trials, sNfL decreases broadly in line with demonstrated treatment efficacies (Bittner et al., 2020; Delcoigne et al., 2020). Furthermore, higher sNfL is predictive of poorer future clinical outcomes at every stage. NfL concentrations are elevated 6 years prior to the clinical onset of MS (Bjornevik et al., 2020). In clinically isolated syndromes, higher sNfL independently predicted faster conversion to clinically definite MS (Disanto et al., 2016). Following diagnosis, higher sNfL has been associated with short and long term poorer outcomes, including relapses, EDSS score progression including progression independent of relapse-activity, clinical conversion to a progressive phenotype, poorer cognitive measures as well as both MRI lesion activity and atrophy (Disanto et al., 2017; Barro et al., 2018; Chitnis et al., 2018; Siller et al., 2018; Cantó et al., 2019; Lorscheider and Benkert, 2020; Thebault et al., 2020a). Some experts consider this test to be on the cusp of widespread clinical adoption (Leppert and Kuhle, 2019), while others remain skeptical (Javed and Stankiewicz, 2020). As early adopters of sNfL testing in MS, in this review, we summarize what we see are key unknowns before the test could and should be deployed in routine clinical practice.

### Hurdles to Widespread Clinical Translation of sNfL

**FIGURE 1:**
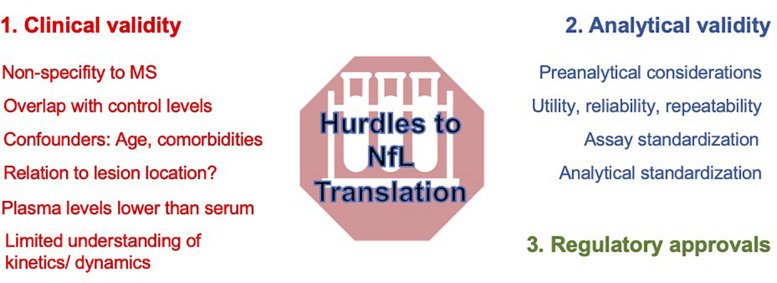
Barriers to the clinical translation of sNfL in 2021.

Despite showing great promise, there are a number of issues relating to sNfL that must be either overcome or at least better appreciated before it can be considered part of the routine armamentarium of MS care (Figure 1). Challenges can be summarized in terms of analytical validity (test performance for sNfL) and clinical validity (sNfL performance as a surrogate of MS-related clinical outcomes of interest). Regulatory approvals of the test are contingent on both components being satisfactorily being met.

## Clinical Validity

sNfL is not specific for MS pathology. While there is an elegance to this, as both inflammatory and neurodegenerative activity in MS are summarized in a single marker, this means sNfL is not a diagnostic marker in MS. Unlike MRI, where new lesions have a characteristic appearance and can be specially correlated with clinical signs and symptoms, sNfL is agnostic of the underlying pathological process causing neuronal loss. Thus, if an individual patient’s concentration is found to be high or rising, neurologists need to carefully consider confounders such as age and comorbidities (discussed in detail below) and may need to order additional testing such as MRI to clarify the situation.

Furthermore, there is significant overlap in sNfL in MS patients and healthy controls, even including cohorts of patients with the most aggressive forms of MS (Thebault et al., 2019). Owing to the relapsing remitting nature of the condition clinically in many, at any given time, the majority of MS patients might be expected to have similar sNfL to healthy controls (Barro et al., 2018). Concentrations frequently fall in an intermediate/gray zone. Thus, while the correlation between sNfL and important MS outcomes in cross-sectional studies is remarkable, interpretation of individual sNfL concentrations in clinical decision making remains challenging. This again highlights the need for careful consideration or even adjustment for the principal clinical confounders.

### Age

Age is the principal physiologic determinant of sNfL. This is likely attributable to the cumulative effects of subclinical pathologies, such as white matter disease causing accelerated neuronal loss (Khalil et al., 2020). There is a moderate association between sNfL and age in both healthy controls and MS patients alike, with typical r values ranging from 0.6 to 0.7 (Khalil et al., 2020) and an increase in adult control sNfL levels of 2.2% per yer of age (Disanto et al., 2017; Barro et al., 2018). In healthy controls, there is an inflection around the age of 60, after which subsequent age-related sNfL increases accelerate, as does inter-individual variability within a given age cohort (Khalil et al., 2020).

There are two possible solutions to this problem. Firstly, patients could serve as their own baseline, using concentrations obtained during a stable period of remission as the comparator for subsequent serial measurements. This could not only account for age but also other commodities which we outline in the sections below. Using such a technique in a prospective observational cohort of 15 MS patients sampled during alemtuzumab treatment, one study found that sNfL “peaks” (>3 standard deviations above steady state concentrations) were associated with clinical and MRI activity in the majority of cases (Akgün et al., 2019). The downside to this approach is that it requires baseline measurement(s) during a preceding period of stability to serve as a subsequent longitudinal benchmark: this is contentious to define and difficult or impossible to obtain early on in the most active patients who would benefit the most from close monitoring. In this situation, a lack of reduction in sNfL following treatment initiation is itself meaningful (Huss et al., 2020) and could be used to guide escalation.

Alternatively, or perhaps in conjunction, others have adjusted for age by comparison to normative datasets from healthy controls. This approach is principally limited by the availability of large biobanks of healthy control sera required to generate such data. The Swiss group based in Basel has been particularly successful, initially presenting patient data in relation to percentiles of healthy control concentrations (Barro et al., 2018), but more recently and statistically rigorously as *z*-scores of log-normalized sNfL (Yaldizli et al., 2020). The availability of such normative datasets as well as ability of local laboratories to apply an age-adjustment factor is undetermined. Nonetheless, a relatively simple age adjustment which can be calculated for both a single measurement as well as serial measurement means is a significant step toward being able to use sNfL measurement to follow individual patents.

### Confounding Effects of Other Neurological and Non-neurological Comorbidities

Extensively reviewed elsewhere (Khalil et al., 2018; Barro et al., 2020), higher sNfL is seen in many central and peripheral nervous system diseases that involve neuroaxonal injury including neurodegenerative conditions (Forgrave et al., 2019), stroke (Nielsen et al., 2020- plasma concentrations), and peripheral neuropathies (Altmann et al., 2020). Analogous to troponin in cardiac disease, clinical context is required. Fortunately, many alternate explanations of an elevated sNfL are usually clinically apparent or uncommon in the demographic of MS patients requiring active surveillance. More troubling however is the increase in sNfL seen following even mild traumatic brain injury. Here concentrations increase acutely, are predictive of the severity of injury, and remain elevated for several years after the injury (Shahim et al., 2020). High risk groups include military personnel (Boutté et al., 2019) and athletes (Shahim et al., 2018- plasma concentrations). In the context of MS and superimposed head injury, it may be difficult to attribute concentrations or dynamic changes to one pathology or the other.

Iatrogenic causes of sNfL elevation have also been identified. In a cohort of patients over 60 years old serially sampled after non-neurological surgeries requiring general anesthesia (mostly arthroscopies), concentrations increased by 67% and remained elevated beyond 48 h (Evered et al., 2018). An important consideration for MS patients is the possible effects of lumbar puncture: in Macaque monkeys, a lumbar puncture in the preceding 2–3 weeks increased median sNfL by 162% (Boehnke et al., 2020). Although lumbar punctures are generally infrequent events for most MS patients, much of our current understanding of sNfL is derived from intensively investigated cohorts of patients undergoing treatments, many of which underwent frequent lumbar puncture, a possible confounder of concern. Thus, appropriate timing of blood collection is exceedingly important for correct interpretation and can mitigate the potential for misinterpretation of results.

MS patients can also be at risk for other neurological complications that cause sNfL concentrations to rise. For instance, a 10-fold increase was noted at the time of onset of natalizumab-induced progressive multifocal leukoencephalopathy (Dalla Costa et al., 2019). In a cohort of patients undergoing ablative hemopoietic stem cell transplantation for aggressive MS, transient increases in the first year after the treatment reflected chemotherapeutic toxicity (Thebault et al., 2020c). Nonetheless, we feel that the identification of a rapidly rising NfL in these situations could be a useful warning signal to trigger a reassessment and additional investigations to identify the cause, or switch therapy.

Non-neurological conditions are also known to affect sNfL and need to be considered in any comorbid patient. BMI has been shown to have an effect on the sNfL, likely due to an increase in volume of distribution, where every 1 kg/m^2^ rise in body mass index, sNfL decreases by 0.02 pg/ml (Manouchehrinia et al., 2020). Data from the stroke literature suggests that cardiovascular risk factors including hypertension and poor glycemic control and perhaps renal function are also associated with higher sNfL (Korley et al., 2019). Similar to the proposed explanations for increased concentrations with age, these associations could be driven by comorbid but clinically silent white matter disease (Khalil et al., 2020). Renal function may also be important for NfL clearance as a cause for higher measurements in these patients (Akamine et al., 2020).

### Other Physiologic Considerations

Our understanding of the pathophysiologic processes surrounding NfL release, distribution and metabolism are incomplete. This is illustrated by the correlation of serum and CSF concentrations, which typically has an *r*-value of 0.7–0.8 (e.g., Thebault et al., 2019). This equates to about 50% of sNfL being directly attributable to CSF NfL concentrations. Once NfL leaves axons and enters the extracellular space, it is not known what proportion is drained by lymphatic routes vs. direct drainage into CSF. Both individual and dynamic differences in these routes vary in a manner that could impact sNfL. In the CSF, there could be regional variation in NfL correlation, for example in the cul-de-sac of the lumbar cistern where CSF sampling occurs. Blood brain barrier permeability itself may be a confounder; NfL quotient in serum compared to CSF could be selectively increased following periods of inflammation such as that seen in MS relapse, positively skewing serum measures. However recent studies on this topic in MS patients present conflicting results (Kalm et al., 2017; Engel et al., 2020; Uher et al., 2020a). The diurnal timing of blood collection may also be an important consideration; in a study of 15 healthy males, one group found a more than 10% increase in plasma concentrations of NfL in the morning compared to the evening, although were surprised to find that elevation was not seen following acute sleep deprivation (Benedict et al., 2020). A hypothetical explanation for this diurnal variation proposed by the authors is that synaptic pruning in sleep may alter NfL kinetics.

Once NfL enters the blood, there are other physiologic considerations. One such possible confounder is existence of anti-NfL antibodies found in many MS patients (Silber et al., 2002). While the pathogenic potential of these antibodies is debatable, the presence or absence of these antibodies could alter peripheral NfL clearance.

Related to the physiologic kinetics of NfL distribution and clearance, the half-life of sNfL is a key consideration with implications on the frequency of disease activity monitoring. In a longitudinal study of NfL before and after intrathecal catheter insertion, NfL in both CSF and serum peaked at 1 month post-surgery, returning to baseline after 6–9 months (Bergman et al., 2016). In longitudinally sampled MS patients around the time of relapse, sNfL increased 5 months before, peaked at clinical onset, and recovered within 4–5 months (Akgün et al., 2019). In another observational cohort of 94 patients enrolled in the Comprehensive Longitudinal Investigation of Multiple Sclerosis at the Brigham and Women’s Hospital (CLIMB) study, sNfL was elevated by one third in a 3 month window around gadolinium (Gd) enhancing lesions compared to remission samples (Rosso et al., 2020). Thus, while some possible individual influences of sNfL kinetics remain ill-defined, many groups are now selecting testing frequencies in the 3–6 month range for MS disease activity monitoring.

### Possible Importance of MS Lesion Location

It is the authors’ opinion that lesion location may be an important consideration in the interpretation of sNfL. To date, all studies have compared sNfL concentrations to total whole brain lesion volumes on MRI, and identified this to be one of the most consistent associations of sNfL. However, a large lesion in the right frontal lobe would likely result in a very significant elevation in sNfL conceivably with minimal appreciable disability. Conversely a small lesion affecting key brainstem structures may result in a smaller sNfL rise but significant long-term disability. Additionally, we speculate that other factors such as axon density in different brain and spine regions could be important determinants of the quantitively rise in sNfL in response to a given lesion.

## Analytical Validity

### Preanalytical Considerations

Variations in sample acquisition, transport, processing and storage prior to protein quantification are important preanalytical confounders for many blood biomarkers. Although serum and plasma neurofilament levels are very strongly correlated (*r* = 0.96, Sejbaek et al., 2019), plasma concentrations are around 25% lower than paired serum concentrations, highlighting the need to standardize blood measurements to a single specimen type. For this reason in this review we have chosen to focus on serum as the more prevalent and studied blood biofluid to promote comparability and utility. Otherwise, sNfL has shown good stability over multiple freeze-thaw cycles and prolonged exposure to room temperature (e.g., Hviid et al., 2020, reviewed by Table 1 in Barro et al., 2020).

### Assay Standardization

Much of the focus on sNfL in recent years is directly attributable to development of a clinical immunoassay platform capable of detecting the low concentrations in blood. The Single Molecule Array (SiMoA) has transformed NfL from a CSF-only research-marker of merit to its current status on the verge of clinical translation in blood (Kuhle et al., 2016a). Comparison of traditional ELISA with electrochemiluminescence and SiMoA demonstrated the superiority of SiMoA with an analytical sensitivity of 0.62 pg/mL compared with 15.6 pg/mL electrochemiluminescence and 78.0 pg/mL for enzyme linked immunosorbent assay (Kuhle et al., 2016a). This increased sensitivity of SiMoA is able to detect sNfL in 100% of healthy individuals. The SiMoA assay uses a unique ELISA method of detecting very low concentration analytes (Rissin et al., 2010). Briefly, antibodies are linked to a solid surface as in a traditional ELISA, however the SiMoA assay utilizes microbeads 2.7 μm in size that individually fit into a microwell array. When measuring very low concentration analytes (subfemtomolar concentrations), the antigen-bead ratio is approximately 1:1 and follow a Poisson distribution. This distribution suggests that beads carry either a single immunocomplex or none, and with very low analyte concentrations only 1–2% of beads carry an immunocomplex. Detection of such low concentrations is not possible through routine enzymatic methods. To accomplish detection, each individual bead is loaded into a single microwell which can be “digitally” counted. Detection is through fluorescent labeling of immunocomplexes which is sensitive enough to measure a single immunocomplex on a single bead. In this way, the number of beads are counted and quantitated against a standard curve, allowing extremely low analyte concentrations to be reliably measured.

Use of the SiMoA assay has facilitated the measurement of NfL in blood and allowed much of the research in MS. The initial NfL assay developed for the SiMoA assay used a home-brew method developed by the Basel group (Kuhle et al., 2016a). They used monoclonal NfL antibodies developed by Umam Diagnostics (47:3 and 2:1, subsequently purchased by Quanterix) along with bovine NfL calibrators. The majority of the early studies were completed using the home-brew assay. More recent studies use the commercially available Quanterix NF-light^TM^ assay kit which uses recombinant human (rhuman) NfL calibrators. It is important for investigators and clinicians to recognize which assay has been used, as that there is a significant positive bias (5:1) of the home-brew assay relative to the commercial NF-light^TM^ assay (Hendricks et al., 2019).

These assay differences highlights the need for assay standardization, and the role of multi-site validation to inform reproducibility and create standardization protocols. In one such international validation effort of the Quanterix NF-light^TM^ assay that sought to assess a variety of analytical outcomes including instrument qualification, precision, level of detection and level of quantification, parallelism and proficiency, the assay performed well across 17 sites with intra- and inter-assay coefficients varying less than 6 and 9%, respectively (Kuhle et al., 2018). However, as interest in this biomarker increases, several other assay platforms are now showing near-equivalent dynamic range and level of detectability. While this competition will drive down setup and testing costs to increase availability, careful work will be needed to confirm inter-platform equivalence.

### Data Analysis and Clinical Reporting

Of the published data available, there is significant variation in data analysis methodologies, limiting inter-study comparability or subsequent meta-analyses. Groups have variably reported measures of central tendency with the mean, median, and geomean. Subsequently reported statistics have included a mixture of parametric and non-parametric techniques, sometimes inappropriately deployed. In our experience, sNfL concentrations are distributed logarithmically. Therefore, comparisons of raw conentrations are constrained to non-parametric techniques, whereas more powerful parametric statistics are possible following Ln/log transformation. Preliminary data from the Basel group (Yaldizli et al., 2020) takes this one step further as they generated age-adjusted z-scores of log transformed data. Although use of *z*-scores may be the most appropriate technique for dealing with age-related increases in sNfL, it may pose challenges for reporting from clinical diagnostic laboratories. Similarly, future data analysis challenges are to determine the most meaningful and clinically deployable measure of sNfL change when trending values. Currently, it is unclear if the raw number alone, reported with reference to an age adjusted population, is important or if an absolute or relative increase is most clinically relevant. Regardless, with every statistical manipulation beyond simple reporting of raw values and cut-offs, may cause implementation hurdles in the clinical diagnostic laboratory and may become practically challenging in a real-world clinical setting.

## A Current Role for sNfL in MS?

We already know that as an adjunctive measure in MS, high or increasing concentrations of sNfL are associated with relapses, EDSS worsening, lesions on MRI scans and atrophy of both the brain and spinal cord. Conversely, serially low sNfL is reassuring. Yet many see the limitations and unknowns relating to the precise interpretation of individual sNfL concentrations so problematic that the marker is “not ready for prime time” (Javed and Stankiewicz, 2020). However, it is the authors’ opinion that the clinical translation of sNfL need not be so black or white. To demand stringent criteria for clinical translation not only ignores the rapidly accumulating body of evidence that already indicates utility for the marker but also seems like a double standard. Neurologists have long tried to use the accurate, often machine generated changes on serial MRI scanning in clinical trials to estimate disease change in their MS patients, only to be challenged with inaccuracies in real life (as opposed to carefully regimented clinical trial MRI studies) due to malalignment, different MRI sequences, different scanners or simply differences in the quality of imaging. Despite this, MRI has become the gold standard non-clinical means for measuring disease in MS. This has not stopped clinicians from making interpretations from serial scans to inform on treatment decisions.

**FIGURE 2:**
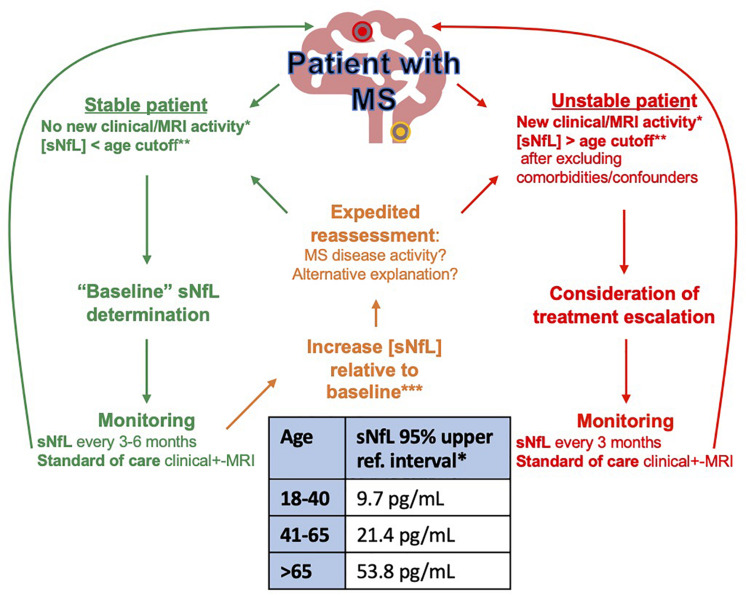
Proposed algorithm for NfL monitoring in MS. *Clinical or MRI disease activity: New relapses, EDSS worsening, New/enlarging MRI lesion. **sNfL 95% age-dependent upper reference interval calculated on SiMOA HD1 instrument using Quanterix NF-light^TM^ (Hviid et al., 2020). ***The increase in sNfL from baseline that best denotes impending disease activity that should prompt further action is still to be determined. Preliminary data from 58 patients with MS followed every 3 months over 1 year suggests that a doubling of sNfL from baseline is associated with a 2.2 × relative risk of relapse (Thebault, Unpublished observation).

It is our opinion that with a good appreciation of the shortcomings and pitfalls, individual patient sNfL concentrations are already a helpful adjunct to clinical practice. In Figure 2 we propose how sNfL can be incorporated into clinical practice as it currently stands: an imperfect marker, that should never be interpreted in isolation. We find it a helpful adjunctive tool and a useful trigger for expedited reassessment when unexpectedly high or rising. Better age dependent based on parametric *z*-score cutoffs (rather than non-parametric percentile cutoffs) are imminently and eagerly awaited. As concerns of clinical validity are better understood if not accounted for and the analytical validity is further established, we hope this marker will be further incorporated into the standard of care. Enabled by less constraining approval processes for clinical use in some jurisdictions, some centers such as our own are already using this test routinely in the MS clinic.

## sNfL, a Future Standard of MS Care?

**FIGURE 3:**
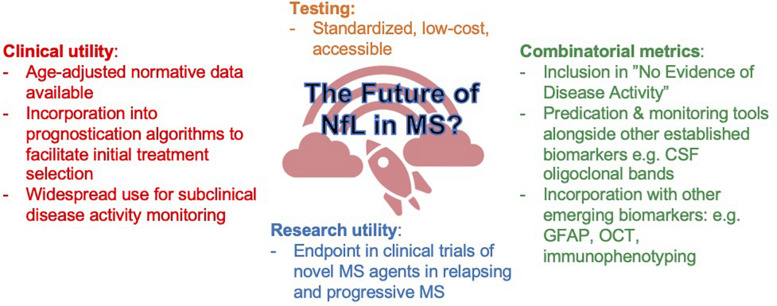
Aspirational predictions of sNfL in the next 5–10 years.

Analogous to the implementation of MRI in routine disease activity monitoring in MS, neurologists will require some education on how to correctly interpret sNfL and incorporate it into routine clinical practice. Similar to MRI, using sNfL may require the establishment of a “baseline” from whence future changes can be referenced and size changes be interpreted. The establishment of better of age-adjusted normative datasets (reference intervals) and biological variation (reference change values) will be a vital step in further individualizing sNfL group level associations. As with many new technologies, the cost of NfL testing itself remains high; competition will help reduce these costs but also present new issues relating to inter-assay comparability (Figure 3).

sNfL in patients with early-stage disease could be incorporated into prognostic models and aid initial treatment selection. Serial measurements, for instance every 3 months, could be useful to monitor for subclinical disease activity both on or off treatment. Here, increasing sNfL would be an objective trigger for neurologists to consider expedited clinical and MRI reassessment, and serially low or stable sNfL would be reassuring (Uher et al., 2020b). There is already substantial evidence for sNfL to be included in future definitions of “no evidence of disease activity.” Thus, through more refined initial treatment selection and closer disease activity monitoring, we think sNfL could have the power to modify the trajectory of MS for the better and improve outcomes. Furthermore, sNfL could reduce current costs by optimizing utilization of MRI, where annual scans for all clinically stable patients is not only expensive but also unfeasible in many settings and could be perhaps better targeted to patients with high or rising sNfL. While the role of sNfL as a clinically useful marker in progressive MS is less clear, this remains a key area of need where clinical responsiveness can be more difficult to quantify. Finally, the potential of sNfL may be augmented by the inclusion of additional markers into combinatorial metrics. While sNfL represents an important first step in a biomarker-driven personalization of MS care, it certainly will not be the last.

## Author Contributions

ST conceived the study, performed the literature review drafted, and edited the manuscript. RB, CR, and HM drafted the manuscript. MF conceived the study, drafted, and edited the manuscript. All authors contributed to the article and approved the submitted version.

## Conflict of Interest

The authors declare that the research was conducted in the absence of any commercial or financial relationships that could be construed as a potential conflict of interest.
